# Supplementary material for: Identification of photoperiod-induced specific miRNAs in the adrenal glands of Sunite sheep (Ovis aries)
Source: Front Vet Sci. 2022 Jul 22;9:888207. doi: 10.3389/fvets.2022.888207 (PMC9354845; doi:10.3389/fvets.2022.888207)
Supplement: Supplementary file 1 [file Data_Sheet_1.ZIP › Supplementary file/miRDeep2 file.zip!/miRDeep2 file/Novel_Structure/SP42A1/Novel_608.pdf]

Provisional ID	:	NC_019472.2_18786
Score total	:	21.9
Score for star read(s)	:	3.9
Score for read counts	:	14.9
Score for mfe	:	1.5
Score for randfold	:	1.6
Score for cons. seed	:	
Total read count	:	41
Mature read count	:	40
Loop read count	:	0
Star read count	:	1

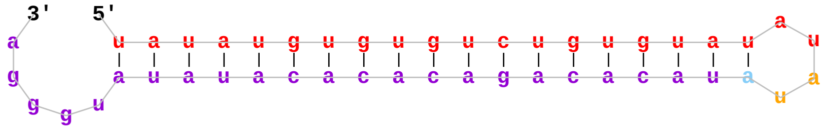

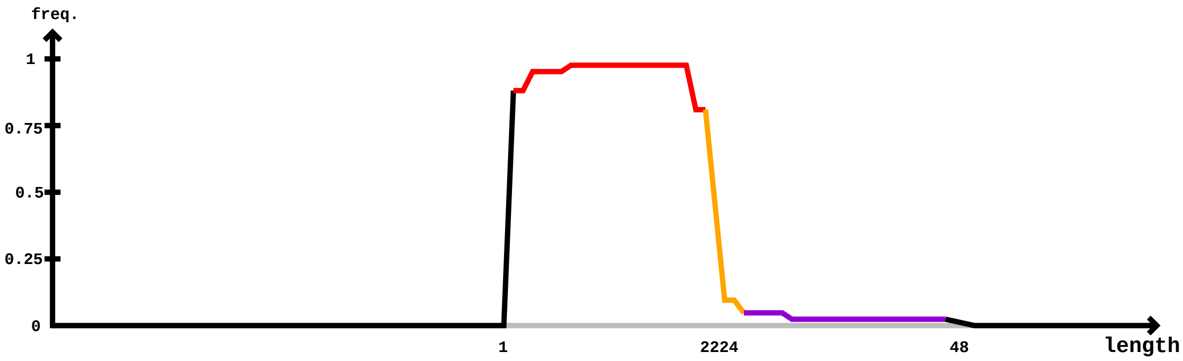

Mature Star

```
5'- uuuucccauggggaaaaaaaaaaacuagcaggauccgucaauacaguauaugugugucuguguauauauauacacagacacacauauaugggaaguacauuauuuauauauua  -3'   obs
    uuuucccauggggaaaaaaaaaaacuagcaggauccgucaauacaguauaugugugucuguguauauauauacacagacacacauauaugggaaguacauuauuuauauauua         exp
    (((((((...)))))))..........................(.(((((((((((((((((((((....))))))))))))))))))))).)....................    reads      mm         sample
    ..............................................uauauguguguAuguguau................................................    7          1          seq
    ..............................................uauauguguguAuguguauau..............................................    21         1          seq
    ..............................................uauauguguguAuguguauaua.............................................    9          1          seq
    ................................................uauguguguAuguguauauaua...........................................    3          1          seq
    ....................................................ugugucuguguauauauauacaU......................................    1          1          seq
    ......................................................................uacacagacacacauauauggga....................    1          0          seq
```
